# Outcomes from Early Experience with Laparoscopic Inguinal Hernia Repair Versus Open Technique: Navigating the learning curve

**DOI:** 10.18295/squmj.5.2023.037

**Published:** 2024-05-27

**Authors:** MA Raajeshwaren, Chellappa Vijayakumar, Souradeep Dutta, Vishnu PN Ramakrishnaiah

**Affiliations:** Department of Surgery, Jawaharlal Institute of Postgraduate Medical Education and Research, Puducherry, India

**Keywords:** Hernia, Inguinal Hernia, Laparoscopy, India

## Abstract

**Objectives:**

This study aimed to evaluate the outcomes of laparoscopic inguinal hernia repair (LIHR) regarding postoperative pain, recurrence rates, duration of hospital stay and other postoperative outcomes within the context of a tertiary care teaching hospital in South India, and the initial experience of laparoscopic repairs. The current consensus in the literature often suggests LIHR as superior to open inguinal hernia repair (OIHR).

**Methods:**

This single-centre, retrospective, observational study was conducted at the Jawaharlal Institute of Postgraduate Education and Research, Puducherry, India, from January 2011 to September 2020. All patients who underwent elective OIHR and LIHR were included. Data on the patients demographics, comorbidities, hernia type, mesh characteristics, surgery duration, hospital stay and immediate postoperative complications were collected and analysed.

**Results:**

A total of 2,690 OIHR and 158 LIHR cases were identified. The demographic profiles, hospital stay and complication rates were similar in both groups. However, surgical site infection was present exclusively in the OIHR group (3.55% versus 0.0%; *P* <0.05). The timeline for returning to normal activities was statistically shorter for the LIHR group (6 versus 8 days; *P* <0.05). The most frequent immediate complication in the LIHR group was subcutaneous emphysema (6.54% versus 0.0%; *P* <0.05). Recurrence (9.23% versus 3.61%; *P* = 0.09) and chronic pain (41.53% versus 13.55%; *P* <0.05) were higher in the LIHR group.

**Conclusion:**

Lower recurrence and chronic pain rates were observed with OIHR in the initial experience with LIHR in the hospital. However, LIHR had significant advantages concerning faster patient recovery and lower rates of surgical site infections. While the results contribute an interesting deviation from the standard narrative, they should be interpreted within the context of a learning curve associated with the early experience of the research team with LIHR.

**Advances in Knowledge**

- *In the initial phase of the adoption of laparoscopy in inguinal hernia repair practice, recurrence and chronic pain rates were found to be higher compared to open repair.*
- *Laparoscopic inguinal hernia repair (LHIR) patients showed significantly lower surgical site infection rates and a faster return to normal activities than open inguinal hernia repair patients. The immediate complication most observed in LIHR was subcutaneous emphysema.*
- *Study results deviated from the typical narrative favouring LIHR, potentially reflecting the learning curve associated with the implementation of new surgical techniques.*

**Application to Patient Care**

- *The findings emphasise the importance of comprehensive training in LIHR to potentially reduce recurrence and chronic pain rates over time. Recognising the role of the learning curve in early LIHR adoption can guide the development of educational and support mechanisms for surgical teams.*
- *Knowledge of the lower surgical site infection rate and faster recovery associated with LIHR can inform patient-physician discussions and decision-making about surgical options.*

Inguinal hernias constitute a significant proportion of routine clinical encounters, representing approximately 75% of all abdominal hernias.^1^ Globally, inguinal hernia repair (IHR) is an extensively performed surgical procedure which is done on upwards of 20 million people.^2^ While surgery serves as the definitive treatment, the choice between laparoscopic and open techniques remains a topic of ongoing discussion. Contemporary studies suggest a decrease in postoperative pain following laparoscopic IHR (LIHR) and a higher incidence of surgical site infections (SSI) associated with open IHRs (OIHR).^3^^,^^4^ Notably, patient recovery following LIHR tends to be more expedient.

A significant challenge with LIHR is its comparatively steep learning curve, underscoring the importance of surgical techniques in mitigating complications. Standardisation of LIHR is instrumental in reducing recurrence rates, expediting recovery and decreasing postoperative complications such as pain and SSI. The surgeon’s experience, thus, holds a critical influence on surgical outcomes.^5^^,^^6^ Recurrence rates with LIHR have been shown to decline with increasing surgeon experience and volume of hernia repairs performed.^7^ Against this backdrop, the current study aimed to analyse and compare the recurrence rates among patients undergoing LIHR and OIHR, specifically within the context of the early experience with laparoscopic techniques at the department of Surgery at the Jawaharlal Institute of Postgraduate Medical Education and Research, Puducherry, India.

The researchers hypothesise that LIHRs may demonstrate differences in outcomes such as recurrence rates, postoperative complications and chronic pain, compared to OIHR. Furthermore, the research posits that the experience level of the surgeon and the surgical approach may play a significant role in determining these outcomes.

## Methods

This single-centre, retrospective, observational study took place at the Jawaharlal Institute of Postgraduate Education and Research, Puducherry, India, and included all patients who underwent elective OIHR and LIHR from January 2011 to September 2020 based on the hospital medical records. This study was conducted from July 2020 to April 2021 and the patients were interviewed over the telephone because of COVID-19 restrictions. The study excluded IHR done under local anaesthesia, laparoscopy converted open repair, hernia with hydrocele, giant hernia with a sliding component, scrotal abdomen and additional procedures such as bowel or omental resection.

This study also excluded emergency hernia repair (inguinoscrotal approach), recurrent hernia repair, bilateral hernia and femoral hernia repair. This study recorded baseline demographic parameters, intraoperative and immediate postoperative outcomes such as duration of hospital stay, intensive care unit stays, surgical complications and reoperations. This study identified immediate complications such as paralytic ileus, haematoma, seroma, SSI and urinary retention in the hospital medical records. Telephone interviews were conducted with each patient to help assess late postoperative outcomes such as recurrence, chronic pain and their characteristics. This telephone interview was the single point of contact between the patient and the investigator.

Recurrence was recorded as the appearance of the inguinal swelling in the previously operated site. This recurrence was graded as per the patient’s words—being smaller, bigger or the same size as the previous swelling before surgery. The precipitating factors for the recurrence, such as heavy weightlifting and chronic cough, were also recorded. Chronic pain was recorded as pain at rest and pain with movement. The frequency was assessed as no pain, rare pain, once or twice a week and continuous pain. The intensity of pain was graded as mild (tolerable pain not affecting daily routine), moderate (needed rest from the daily routine for relief) and severe (required painkillers for pain relief and affected daily routine). The preoperative, intraoperative and postoperative parameters which influenced the primary and secondary outcomes were noted for analysis.

The expertise of the surgeons who operated the LIHR was graded based on their years of experience in LIHR. The surgeons who had less than 3 years of experience were graded as level I. Those with 4–6 years of experience in LIHR were graded as level II, and those with more than 6 years of experience in LIHR were graded as level III. Based on this, the outcomes were analysed. Sub-group analysis of LIHR with robotic IHR was done for postoperative outcomes.

The sample size was calculated using the OpenEpi software, Version 3.1 (Emory University, Atlanta, Georgia, USA)—keeping the proportion of group 1 - LIHR patients with recurrence of hernia as 3.4% (exposed with the outcome) and the same in group 2 - OIHR as 5.2% (unexposed with the outcome), with 80% power and an alpha of 5%—as 1,652 in each group. From the medical record review, it was found that only 158 cases of laparoscopic hernia repairs for primary unilateral hernias had been done during the study period, and hence, a sample size of 1,652 was not achievable. Therefore, after inclusion and exclusion criteria for the above inguinal hernia repairs were performed, a total of 107 and 1,898 cases were analysed in the LIHR and OHR groups, respectively. Since there was a massive difference in the total number of cases between the two groups, the total number of cases taken was in the ratio 1:5 (i.e. 107 versus 535). This was considered as there was no significant difference in the *P* value for cases more than 4 times the control.

Statistical analysis was done using the Statistical Package for the Social Sciences (SPSS), Version 19.0 (IBM Corp, Armonk, New York, USA). The Mann-Whitney U test was used. All categorical variables were expressed as proportions. They were analysed appropriately with the Chi-squared test or Fischer’s exact test based on the normality evaluated by the Shapiro-Wilk test. The logistic regression analysis was done for the primary outcome, i.e. the recurrence. Independent variables were analysed for their association with recurrence and those which had a *P* value of <0.2 were used for multivariate regression.

The odds ratio with 95% confidence interval (CI) and *P* value was summarised and used to interpret the association of independent variables with outcome. A *P* value <0.05 was considered statistically significant. Ethical approval from the Institutional Ethics Committee of the hospital was obtained in 2019 (JIP/IEC/2019/529).

## Results

**Figure 1:**
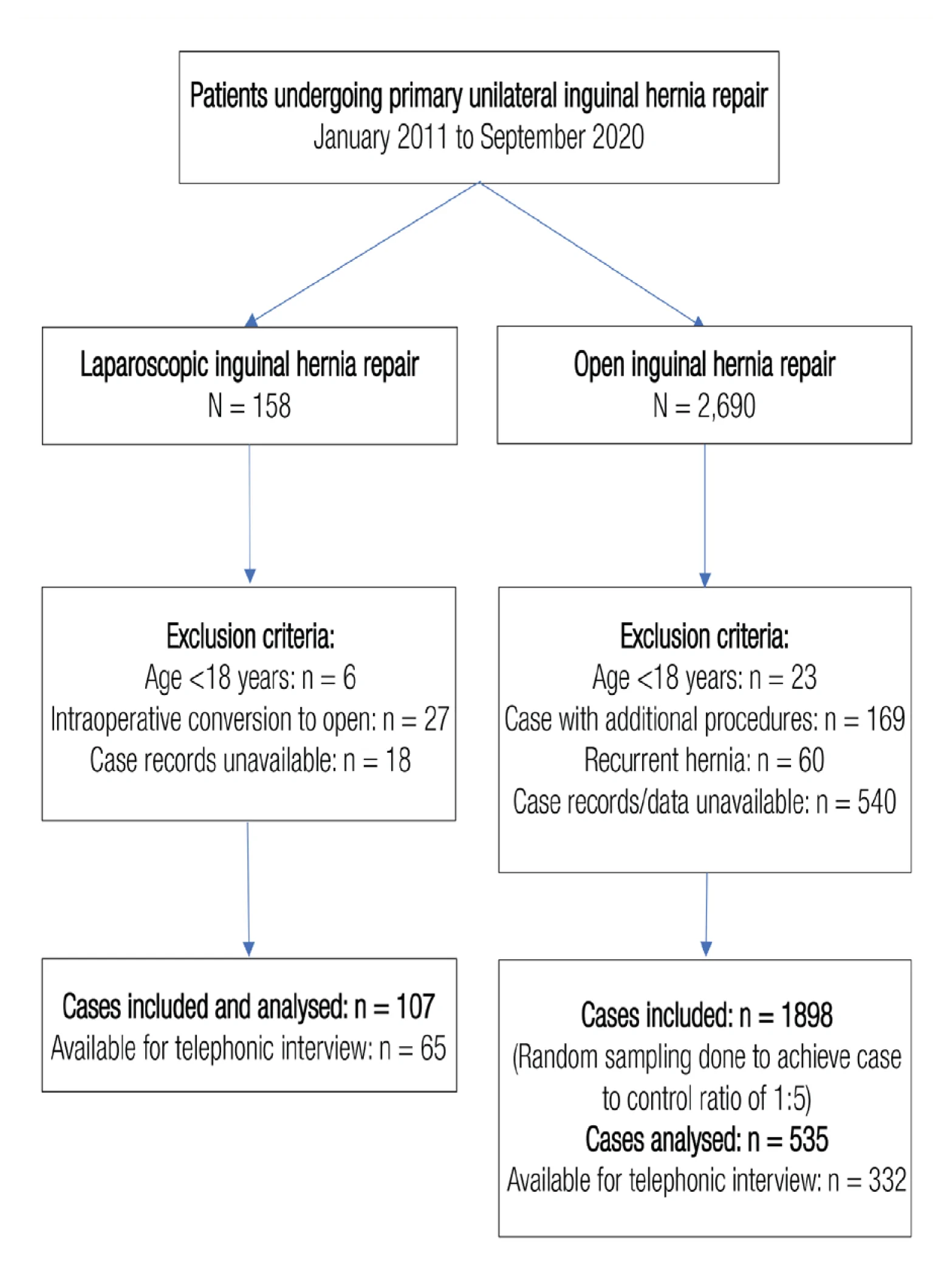
Flowchart showing the included cases in this study.

A total of 158 cases of LIHR and 2,690 cases of OIHR were identified during the study period. Based on exclusion and inclusion criteria, 107 patients were chosen in the LIHR group and 1,898 in the OIHR group. However, given the discrepancy in the number of cases, the researchers analysed only 642 patients (107 in LIHR and 535 in OIHR) who underwent hernia surgery between January 2011 and September 2020 [Figure 1].

Most patients were older than 40 years (61.53%), with a median age of 47 years. The pattern of patient distribution was similar in both groups, except for the OIHR group having a greater proportion of smokers; 6.17% of the OIHR group patients were smokers compared to 2.80% in the LIHR group. The prevalence of benign prostate hypertrophy was similar in both groups (8.41% versus 7.66%; *P* = 0.79). The overall percentage of patients with comorbidities between the groups was similar [Table 1].

The usage of prophylactic antibiotics was subject to the surgeon’s discretion. This difference was statistically significant between the two groups (84.11% versus 69.53%; *P* <0.05). Out of the patients who received antibiotics, the majority of them received ≤3 doses of antibiotics. However, in this study, the usage of antibiotics did not affect SSI (*P* = 0.13). The indirect sac was most commonly identified in both groups accounting for 71.03% in the LIHR and 68.04% in the OIHR group. Approximately 96.82% of the patients in the OIHR used a 15 × 7 cm mesh. The mesh used for the entire cohort of the OIHR was made up of polypropylene. The difference between the groups was statistically significant (*P* <0.05). In the majority of the LIHR group (79.44%) the mesh was fixed using tackers. Almost the entire cohort of the OIHR cases (97.94%) had the mesh fixed with polypropylene sutures.

Most of the patients did not have any content in the hernia sac, majorly due to a reduction of the content preoperatively. The most commonly encountered content intraoperatively was omentum accounting for (17.76% and 22.24%) the LIHR and OIHR cases, respectively. The distal sac was reduced (66.36% versus 15.70%) primarily in LIHR, but it was transfixed (15.89% versus 74.02%) predominantly in the OIHR. The duration of the procedure was more for the LIHR than the OIHR. It was very clearly established that an open hernia needed less time to operate and the difference was statistically significant (150 versus 75 minutes; *P* <0.05). The median duration of hospital stay was also similar in both groups, i.e. 3 days with an Inter Quartile Range (IQR) of 3–4 days in the LIHR group and 2–3 days in the OIHR group (*P* <0.05) [Table 2].

None of the patients in the LIHR group developed SSI. This finding was statistically significant (*P* <0.05). A total of 12 patients had scrotal edema following OIHR surgery, while none in the LIHR group developed it (*P* <0.05). The most encountered immediate complication in the LIHR was subcutaneous emphysema; this was statistically significant (*P* <0.05). This study found that patients who developed SSI were in the OIHR (3.55%) group and not in the LIHR group (0.00%). Urinary retention was similar in both groups in this study [Table 3]. The data on the late postoperative outcomes could be obtained only from 65 and 332 patients, in the LIHR and OIHR groups, respectively, via telephone conversations. In this, the recurrence rate between the two groups was 9.23% (n = 6) in the LIHR group and 3.61% (n = 12) in the OIHR group. The recurrences were significantly more in terms of numbers, but they were not statistically significant (*P* = 0.09). The difference in chronic pain between the groups was statistically significant (41.53% versus 13.55%; *P* <0.05) [Table 4].

The time taken for the patients to do their normal routine activities was 6 days and 8 days for the LIHR and OIHR groups, respectively. The distribution was again a non-normally distributed one with a few outliers in the group. This was mainly due to the development of complications. The 25th percentile was 4 and 6 for LIHR and OIHR groups, while the 75th percentile was 10 for both groups. The difference between the groups was statistically significant (*P* <0.05). The odds of developing chronic pain with movement were 5.28 times more for LIHR with a 95% CI of 2.91–9.59 and, thus, significant. The odds of recurrence were 2.69 times higher for the LIHR group than for the OIHR group. However, the 95% CI was wide (0.97–7.46), which makes it an insignificant value. Similarly, the odds of developing a seroma or chronic pain at rest were 2.61 and 0.83 times for the LIHR group and the OIHR group, respectively. However, the CI was wide (0.23–29.29 and 0.24–2.89, respectively).

The odds of recurrence were higher with diabetes mellitus (DM), followed by time to return to normal activities and SSI. DM, superficial SSI and time to return to normal activities had *P* <0.05. In a multivariate regression analysis, the significant variables in the LIHR group were: history of smoking, presence of DM, duration of the procedure, mesh fixation with tackers, the number of doses of antibiotics, time to return to normal activities and the presence of superficial SSI. This analysis showed that the presence of DM, time to return to normal activities and superficial SSI were factors that had a significant influence on the recurrence of the hernia (adjusted odds ratio = 19.01, 1.16 and 8.15, respectively) [Tables 5 & 6].

Recurrence was found only in 1 out of 26 patients operated by level III surgeons. However, recurrence was reported in 5 out of 40 cases performed by level II and level I surgeons. A total of 9 out of 21 patients operated by level I surgeons developed chronic pain with movement. The same was reported in 9 out of 19 patients for level II surgeons and 9 out of 26 patients in level III surgeons.

## Discussion

Inguinal hernia consistently ranks as a common condition faced in general practice. Surgical interventions such as LIHR and OIHR form the definitive therapeutic approach. In the current study, both techniques shared a similar hospital stay duration averaging approximately 3 days. Importantly, LIHR demonstrated a significantly faster recovery time back to normal activities. Nonetheless, complication rates between the two groups were similar, while recurrence and chronic pain were observed more frequently in the LIHR cohort. These findings, perhaps, could be reflective of the institution’s relative early experience with LIHR as compared to OIHR, suggesting the significance of the surgical learning curve in impacting outcomes.

On examination of the demographic data, it became clear that comorbidity prevalence profoundly impacted postoperative complication development. The current study as well as the study by Ruhl *et al*. found a predominance of patients older than 40.9 years.^8^ Additionally, right-sided hernias were more common, likely due to the later closure of the *processus vaginalis* on this side. Notably, lifestyle factors and comorbidities such as tobacco use, alcohol and DM were implicated in structural remodelling of the inguinal region, thereby increasing the incidence of inguinal hernia.^9^

In the realm of intraoperative parameters, the current study mirrored prior research, showing a greater prevalence of indirect than direct sacs.^10^^,^^11^ The majority of patients had no hernia sac content intraoperatively, mainly due to preoperative reduction efforts. Interestingly, after overcoming the learning curve, surgeons demonstrated no significant differences in operating times between techniques.^12^^,^^13^ Regarding antibiotic prophylaxis, the need for a balance between minimising SSI rates and avoiding unnecessary antibiotic use became evident.^2^^,^^14^

Concerning early postoperative complications, the occurrence of subcutaneous emphysema was higher in the LIHR group, attributed to the nature of gas insufflation during the procedure.^15^–^17^ Post-LIHR urinary retention appeared more common, although robust evidence is lacking.^18^^,^^19^ The return to routine activities was significantly quicker with LIHR, which has been echoed in various studies.^20^^,^^21^ Regarding late postoperative complications, a higher recurrence rate in LIHR was noted, which might be associated with the steep learning curve of this procedure.^2^^,^^22^ While the recurrence rates seemed higher in the LIHR group, the statistical analysis did not find a significant difference. This could be attributed to various factors such as the smaller sample size in the LIHR group, which might have limited the study’s ability to detect a significant difference. Additionally, other confounding factors, such as the learning curve, varying surgical techniques or patient selection, might have influenced recurrence rates. However, risk factors such as DM and wound infection did not significantly affect recurrence rates.^23^^,^^24^ The study findings deviated from the consensus in terms of chronic pain incidence, which was higher with LIHR, aligning with Huerta *et al*.^16^^,^^25^ This departure from the trend may be ascribed to the early experience stage of the institution with laparoscopic techniques for managing inguinal hernias.

In recent years, there has been increasing interest in the integration of artificial intelligence (AI) and deep learning into surgical practice, aimed at enhancing surgical precision, optimising patient outcomes and reducing complications.^26^ While the current study focuses on traditional laparoscopic and open hernia repairs, the evolution of surgery with technological advancements cannot be ignored. It is imperative to acknowledge the potential challenges and benefits of integrating AI into surgical procedures.^26^ As hernia repair techniques continue to evolve, it is crucial to remain updated with the latest technological advancements and their implications.

The current study has several limitations that need to be considered while interpreting the results. First, the retrospective nature of the research inherently carries the risk of information bias, with potential discrepancies in the data recording process over time. The long study period also exposes the analysis to changes in surgical techniques, equipment and postoperative care protocols, all of which could affect outcomes. Second, the marked difference in the sample sizes between the OIHR (n = 2,690) and LIHR (n = 158) groups pose challenges in drawing direct comparisons and could potentially skew the findings. The smaller sample size in the LIHR group could have made the detection of rare complications less likely compared to that in the larger OIHR group. Finally, the grading of surgeon expertise based solely on years of experience in LIHR, though a useful proxy, does not consider other vital factors such as the volume of surgeries performed, specific training and continuous skill upgrades. This grading may overlook nuances in surgical proficiency, as years of experience might not directly correlate with skill or outcomes. Future studies should employ a more comprehensive and objective measure of surgical expertise to further elucidate the role of surgeon skill in patient outcomes.

## Conclusion

The research findings underscore the importance of the surgical learning curve in achieving optimal outcomes in LIHR. While LIHR demonstrated faster recovery times compared to OIHR, it also revealed a higher incidence of recurrence and chronic pain. These trends may be attributed to the institution’s relative early experience with LIHR. Furthermore, the current study highlights the significance of comorbidities and lifestyle factors in hernia development and postoperative complications. Despite the limitations inherent in a retrospective study, this investigation provides valuable insights into the management of inguinal hernias. Future prospective studies with larger cohorts are needed to confirm the study findings and enhance the understanding of LIHR outcomes concerning the learning curve and early experience of surgeons.

## Figures and Tables

**Table 1:** Characteristics of the patients who underwent laparoscopic or open inguinal hernia repair (N = 642)

Characteristic	Group, n (%)		*P* value
	LIHR (n = 107)	OIHR (n = 535)	
**Median age in years**			
Total (IQR)	40 (27–53)	49 (34–61)	<0.05
<40	57 (53.27)	190 (35.51)	
>40	50 (46.73)	345 (64.49)	
**Gender**			
Male	104 (97.20)	522 (97.57)	-
Female	3 (2.80)	13 (2.43)	
**Laterality** *			
Left (n = 239; 37.23%)	43 (40.19)	196 (36.64)	0.48
Right (n = 403; 62.77%)	64 (59.81)	339 (63.36)	
**Risk factor**			
Smoking history	3 (2.80)	33 (6.17)	0.16
Tuberculosis	1 (0.93)	4 (0.75)	1.00
BPH	9 (8.41)	41 (7.66)	0.79
**Comorbidity**			
Diabetes	7 (6.54)	21 (3.93)	0.29
Hypertension	12 (11.21)	44 (8.22)	0.09
CAD	1 (0.93)	17 (3.18)	<0.05
COPD	0 (0.00)	4 (0.75)	<0.05
CKD	0 (0.00)	4 (0.75)	<0.05
Bronchial asthma	0 (0.00)	4 (0.75)	<0.05

LIHR = laparoscopic inguinal hernia repair; OIHR = open inguinal hernia repair; IQR = interquartile range; BPH = benign prostate hypertrophy; CAD = coronary artery disease; COPD = chronic obstructive pulmonary disease; CKD = chronic kidney disease.

* The number of cases is different for these variables because of missing data in the patient’s medical records (these variables were not documented in all study patients’ medical records in both groups. Hence, they were analysed based on the available data).

**Table 2:** Intraoperative parameters of the study population (N = 642)

Variable*	Group, n (%)		*P* value
	LIHR (n = 107)	OIHR (n = 535)	
**Antibiotic prophylaxis**			
Yes	90 (84.11)	372 (69.53)	<0.05
No	10 (9.34)	161 (30.09)	
**Type of hernias**			
Direct sac	25 (23.36)	137 (25.61)	0.10
Indirect sac	76 (71.03)	364 (68.04)	
Both sacs	1 (0.93)	31 (5.79)	
**Size of mesh in cm**			
15 × 7	36 (33.64)	518 (96.82)	<0.05
15 × 10	29 (27.10)	-	
15 × 15	17 (15.89)	1 (0.19)	
Others	9 (8.41)	3 (0.56)	
**Type of mesh**			
Prolene	92 (85.98)	524 (97.94)	<0.05
Polyester	4 (3.74)	0	
Drain	0 (0.00)	3 (0.56)	1.00
**Mesh fixation**			
Tackers	85 (79.44)	-	<0.05
Sutures	1 (0.93)	524 (97.94)	
Clips	1 (0.93)	-	
**Intra-operative conversion of TEP to TAPP**	3 (2.80)	-	
**Content of sac**			
Bowel	5 (4.67)	48 (8.97)	0.24
Omentum	19 (17.76)	119 (22.24)	
Preperitoneal fat	5 (4.67)	9 (1.68)	
No content	69 (64.49)	351 (65.61)	
Other	0 (0.00)	2 (0.37)	
**Distal sac**			
Reduced	71 (66.36)	84 (15.70)	<0.05
Transfixed	17 (15.89)	396 (74.02)	
Excised	3 (2.80)	5 (0.93)	
No sac	2 (1.87)	34 (6.36)	
Ligated	0 (0.00)	1 (0.19)	
Left behind	3 (2.80)	2 (0.37)	
**Median duration of procedure in min (IQR)**	150 (117–182)	75 (60–100)	<0.05
**Median blood loss in mL (IQR)**	30 (20–50)	30 (20–50)	0.30
**Median duration of hospital stay in days (IQR)**	3 (3–4)	3 (2–3)	<0.05
**Patients with ICU stay**	2 (1.87)	3 (0.56)	0.51

LIHR = laparoscopic inguinal hernia repair; OIHR = open inguinal hernia repair; TEP = totally extraperitoneal; TAPP = transabdominal preperitoneal; IQR = interquartile range; ICU = intensive care unit.

* The total number of cases is different for each variable because of missing data in the patient’s medical records (these variables were not documented in all study patients’ medical records in both groups. Hence, they were analysed based on the available data).

**Table 3:** Immediate and early postoperative outcomes of the study population (N = 642)

Variable	Group, n (%)		*P* value
	LIHR (n = 107)	OIHR (n = 535)	
**Immediate postoperative complication**			
Subcutaneous emphysema	7 (6.54)	0 (0.00)	<0.05
Ileus	1 (0.93)	8 (1.49)	0.86
Fever	5 (4.67)	12 (2.24)	0.09
Urinary retention	3 (0.28)	3 (0.56)	0.43
Urinary tract infection	1 (0.93)	2 (0.37)	0.67
Surgical site infection	0 (0.00)	19 (3.55)	<0.05
Superficial	0	(0.00)	14 (2.62)
Deep	0 (0.00)	5 (0.93)	
Scrotal edema	0 (0.00)	12 (2.24)	<0.05
Penile and cord edema	0 (0.00)	3 (0.56)	0.91
Total	15 (14.02)	56 (10.47)	<0.05
**Early postoperative complication**			
Pus discharge	0 (0.00)	19 (3.55)	<0.05
Seroma	1 (0.93)	2 (0.37)	0.41
Haematoma	0 (0.00)	2 (0.37)	1.00
Median time to return to normal activities in days (IQR)	6 (4–10)	8 (6–10)	<0.05

LIHR = laparoscopic inguinal hernia repair; OIHR = open inguinal hernia repair; IQR = interquartile range.

**Table 4:** Late postoperative outcomes among patients available for telephonic interviews (N = 397)

Late postoperative complication	Group, n (%)		*P* value
	LIHR (N = 65)	OIHR (N = 332)	
Recurrence	6 (9.23)	12 (3.61)	0.09
Chronic pain	27 (41.53)	45 (13.55)	<0.05
Pain at rest	3 (4.62)	18 (5.42)	1.00
Pain at	27 (41.54)	39 (11.75)	<0.05
movement			
Port site hernia	0 (0.00)	-	-

LIHR = laparoscopic inguinal hernia repair; OIHR = open inguinal hernia repair.

**Table 5:** Univariate logistic regression of preoperative and intraoperative parameters for recurrence

Variable	OR (95% CI)	*P* value
**Preoperative parameter**		
**Age**	0.99 (0.96–1.02)	0.63
**Risk factors**		
Tuberculosis	(0.00) 0.00	0.99
Benign prostatic hyperplasia	(0.68) 0.08–5.29	0.71
Smoking	(3.57) 0.95–13.34	0.05
Hypertension	(1.73) 0.48–6.25	0.40
**Comorbidity**		
Diabetes mellitus	(5.38) 1.61–17.93	0.006
COPD	(0.00) 0.00	0.99
CAD	(0.00) 0.00	0.99
**Left-sided hernia**	(0.77) 0.28–2.10	0.61
**Intraoperative parameter**		
**LIHR group**	(2.69) 0.97–7.46	0.05
**Content of the sac**		
Omentum	(1.57) 0.52–4.75	0.41
Bowel	(1.36) 0.28–6.49	0.69
**Distal sac**		
Reduced	(0.47) 0.08–2.78	0.41
Transfixed	(0.51) 0.10–2.47	0.40
**Duration of procedure**	(1.00) 0.99–1.01	0.13
**Blood loss**	(1.00) 0.99–1.01	0.75
**Mesh fixation by tackers**	(2.54) 0.86–7.54	0.09
**Postoperative parameter**		
**Antibiotic prophylaxis**	(0.61) 0.23–1.61	0.32
**No. of doses**	(0.68) 0.46–1.00	0.05
**Duration of hospital stay**	(1.15) 0.98–1.38	0.75
**Duration of ICU stay**	(1.73) 0.43–7.019	0.43
**Time to return to normal activities**	(1.15) 1.04–1.26	<0.05
**Surgical site infection superficial**	(17.88) 3.85–83.11	<0.05

OR = odds ratio; CI = confidence interval; COPD = chronic obstructive pulmonary disease; CAD = coronary artery disease; LIHR = laparoscopic inguinal hernia repair; ICU = intensive care unit.

**Table 6:** Multivariate logistic regression table for recurrence with the study population

Variable	OR (95% CI)	*P* value
**LIHR group**	0.00 (0.00)	0.99
**Risk factor**		
Smoker	3.73 (0.79–17.53)	0.09
Diabetes mellitus	19.01 (4.30–84.01)	<0.05
**Duration of procedure**	0.99 (0.98–1.00)	0.12
**No. of antibiotic doses**	0.94 (0.66–1.32)	0.73
**Time to return to normal activities**	1.16 (1.03–1.31)	<0.05
**Superficial SSI**	8.15 (2.1–20.26)	<0.05

OR = odds ratio; CI = confidence interval; LIHR = laparoscopic inguinal hernia repair; SSI = surgical site infection.
